# Impact of Adapting the Abbreviated Injury Scale (AIS)-2005 from AIS-1998 on Injury Severity Scores and Clinical Outcome

**DOI:** 10.3390/ijerph16245033

**Published:** 2019-12-10

**Authors:** Shiun-Yuan Hsu, Shao-Chun Wu, Cheng-Shyuan Rau, Ting-Min Hsieh, Hang-Tsung Liu, Chun-Ying Huang, Sheng-En Chou, Wei-Ti Su, Ching-Hua Hsieh

**Affiliations:** 1 Department of Trauma Surgery, Kaohsiung Chang Gung Memorial Hospital, Chang Gung University and College of Medicine, Kaohsiung 83301, Taiwan; ah.lucy@hotmail.com (S.-Y.H.); hs168hs168@gmail.com (T.-M.H.); htl1688@yahoo.com.tw (H.-T.L.); junyinhaung@yahoo.com.tw (C.-Y.H.); athenechou@gmail.com (S.-E.C.); s101132@adm.cgmh.org.tw (W.-T.S.); 2 Department of Anesthesiology, Kaohsiung Chang Gung Memorial Hospital, Chang Gung University and College of Medicine, Kaohsiung 83301, Taiwan; shaochunwu@gmail.com; 3 Department of Neurosurgery, Kaohsiung Chang Gung Memorial Hospital, Chang Gung University and College of Medicine, Kaohsiung 83301, Taiwan; ersh2127@cloud.cgmh.org.tw; 4 Department of Plastic Surgery, Kaohsiung Chang Gung Memorial Hospital, Chang Gung University and College of Medicine, Kaohsiung 83301, Taiwan

**Keywords:** trauma, Abbreviated Injury Scale (AIS), AIS version, Injury Severity Score (ISS), mortality

## Abstract

Background: In recent years, several versions of the Abbreviated Injury Scale (AIS) were updated and published. It was reported that the codeset in the dictionary of AIS-2005 had significant change from that of AIS-1998. This study was designed to evaluate the potential impact of adapting the AIS-2005 codeset from the AIS-1998 in an established trauma system of a single level I trauma center. The patients’ outcome was measured in different Injury Severity Score (ISS) strata according to the double-coded injuries in a three-year period. Methods: The double-coded injuries sustained by 7520 trauma patients between 1 January, 2016, and 31 December, 2018, in a level I trauma center were used to compare the patient injury characteristics and outcomes between AIS-1998 and AIS-2005 and under different ISS strata, defined as <16 (mild to moderate injury), 16–24 (severe injury), and >24 (critical injury). Results: The mean ISS was significantly lower using AIS-2005 than using AIS-1998 (7.5 ± 6.3 vs. 8.3 ± 7.1, respectively, *p* < 0.001). AIS-2005 scores in the body regions of the head/neck (2.94 ± 1.08 vs. 3.40 ± 1.15, respectively, *p* < 0.001) and extremity (2.19 ± 0.56 vs. 2.24 ± 0.58, respectively, *p* < 0.001), but not in other body regions, were significantly lower than AIS-1998 scores. The critically injured patients (ISS >24), but not severely injured patients or patients with mild-to-moderate injury, coded by AIS-2005 had a significantly higher mortality rate (34.2% vs. 26.2%, respectively, *p* = 0.031) than did patients coded by AIS-1998. The rate of intensive care unit admission was significantly higher for patients in all ISS strata after adapting AIS-2005 as the scoring system than after adapting AIS-1998. Regarding patients with major trauma, which was defined as ISS > 15, the number of patients with major trauma in this study was 17.0% (*n* = 1276) for AIS-1998 and 9.7% (*n* = 733) for AIS-2005. As a consequence, the mortality rate of patients with major trauma was significantly higher in AIS-2005 than in AIS-1998 (15.4% vs. 9.1%, respectively, *p* < 000.1). Conclusions: In this study, we revealed that the adaptation of AIS-2005 from AIS-1998 had resulted in a significant decrease of severity scores in the measurement of the same injuries. The number of head/neck injuries classified as 16–24 was the key difference between AIS-1998 and AIS-2005. Furthermore, critically injured patients who had ISS > 24 coded by AIS-2005 had significantly higher mortality rates than did the patients coded by AIS-1998. This study also indicated that a direct comparison of the measurements that are generated from these two AIS versions can produce misleading results.

## 1. Introduction

The Abbreviated Injury Scale (AIS) was developed in 1971 to measure an anatomy-based injury severity with a simple score to rank specific injuries in trauma patients [1]. The AIS was developed based on several dimensions of severity, including energy dissipation, extent of tissue damage, threat to life, permanent impairment, and treatment period, to assess the severity of the anatomical injury on a six-point ordinal scale ranging from minor (1), moderate (2), serious (3), severe (4), critical (5), to un-survivable injury (6). AIS is the fundamental basis of several severity scoring systems, such as the Injury Severity Score (ISS) [2], the Maximum Abbreviated Injury Score [3], the New Injury Severity Score (NISS) [4,5], and the Exponential Injury Severity Score [5]. The most commonly used prediction model for survival probability of trauma patients, the Trauma and Injury Severity Score (TRISS) [6,7,8], is also calculated based on the ISS.

In recent years, several versions of AIS had been updated and published. AIS-1990, the first to utilize the six-digit identifier, expanded the codes of brain injury and added some modifiers for pediatric injury [9]. AIS-1998 established the guidelines for coding throughout the entire dictionary and improved the specificity for several organ injuries [9]. AIS-2005 made significant changes throughout the dictionary and added over 600 codes [9]. AIS-2008 was the minor revised version of AIS-2005 and included changes in the injury severity level of eight codes, a supplement to the coding instruction, and error correction [10]. Considered the most recent AIS, AIS-2015 completely revised the spinal injury codes and allowed the coding of associated soft tissue injury [9].

The differences between AIS-1990 and AIS-1998 and between AIS-2005 and AIS 2008 were relatively insignificant. One study found that there was no significant change of ISS severity between AIS-1998 and AIS-1990 [11]. Additionally, there were only minor changes between AIS-2005 and AIS-2008 [12,13]. However, there was a significant impact on the measurement of overall injury severity between AIS-2005 and AIS-1998. There was a substantial increase in the codeset size of AIS-2005 (1986 codes) from AIS-1998 (1341 codes) to cover a wider injury spectrum [9,14]. More than 10% of AIS-2005 codes had an assigned injury severity, which was different from the comparable code of AIS-1998.

Two studies had reported that the mean ISS calculated from AIS-2005 was significantly less than that from AIS-1998 [15,16]. Salottolo et al. [16] found a lower calculated ISS, NISS, and maximum AIS with inaccurate subsequent outcome measurement in the studied trauma population. However, some limitations inherent in these studies had been recognized [17]. The ISS, which is an ordinal and highly skewed measure, was assessed using *t*-tests, which was considered statistically inappropriate when analyzing such data [17]. Moreover, there was no discussion regarding the proportion of injuries in each body region with changed severity. Barnes et al. [15] evaluated the proportion of injuries that had changed in their AIS scores in a sample selected from a car occupant injury database in 1994. However, only a small proportion of patients was severely injured. Additionally, the effect of the score changes within each individual body region has not been explored yet [17]. Therefore, this study aimed to evaluate the potential impact of adapting the AIS-2005 codeset from the AIS-1998 in an established trauma system of a single level I trauma center in southern Taiwan using the double-coded injuries in a three-year period with the examination of the effect of these changes on the patients’ outcome measurement in different ISS strata.

## 2. Materials and Methods

### 2.1. Ethics Statement

This study had been approved with a reference number 201900384B0C501 by the Institutional Review Board (IRB) of the Kaohsiung Chang Gung Memorial Hospital. The requirement for informed consent from the patients was waived according to the regulation of IRB.

### 2.2. Study Population

The study double-coded the injuries for all 7520 consecutively admitted trauma patients during a three-year period between 1 January, 2016, and 31 December, 2018, by two certified specialists who had been trained for coding and experienced for three and ten years, respectively, in trauma registry at Taiwan. The study hospital is a level I trauma center in the Kaohsiung city, Taiwan [18,19,20] and serves around three millions patients in southern Taiwan with another two level I trauma centers [21,22,23,24]. About thirty thousand trauma patients had visited the emergency department of this hospital and, among them, there were around 3600 hospitalizations per year. The AIS codes were manually coded according to both AIS-1998 and AIS-2005 to ease the transition between AIS versions. AIS, which scores the severity of injury on a scale of 1–6 for six specified anatomical regions, was prospectively recorded for each AIS version (AIS-1998 and AIS-2005) with corresponding ISS and TRISS calculated. Patient information retrieved included sex, age, hospital length of stay (LOS), admission into the intensive care unit (ICU), and in-hospital mortality.

### 2.3. Statistical Analysis

Patient injury characteristics and outcomes were compared between those coded by AIS-1998 and AIS-2005 and under different ISS strata. The statistical analysis was performed using the Statistical Package for the Social Sciences version 23.0 window software (International Business Machines Corp., Armonk, NY, USA). The categorical variables were analyzed with chi-square analysis or Fisher’s exact analysis. The homogeneity of variance of the continuous variables was first assessed using Levene’s test, followed by one-way analysis of variance with a Games–Howell post hoc test, to evaluate the differences between groups of body regions coded by AIS-1998 and AIS-2005. Continuous data were expressed as mean ± standard deviation. Non-normally distributed data are presented as median ± interquartile range (IQR, Q1–Q3). A *p* value < 0.05 was considered statistically significant.

## 3. Results

### 3.1. Comparison of Injury Characteristics between Abbreviated Injury Scale (AIS)-1998 and AIS-2005

In this study, a total of 7520 hospitalized trauma patients were included. As shown in Table 1, the patient numbers coded with AIS injury are similar in the body regions of the head/neck, face, and abdomen, but not in the body regions of the thorax (AIS-1998, 928 patients; AIS-2005, 930 patients), extremity (AIS-1998, 5162 patients; AIS-2005, 5147 patients), and external organs (AIS-1998, 1070 patients; AIS-2005, 1083 patients). The mean ISS was significantly lower using AIS-2005 than using AIS-1998 (7.5 ± 6.3 vs. 8.3 ± 7.1, respectively, *p* < 0.001). The mortality rate, rate of ICU admission, and median days of hospital LOS of these patients were 1.8% (135 / 7520), 21.2%, and six days, respectively.

### 3.2. Comparison of AIS in Different Body Regions between AIS-1998 and AIS-2005

The mean AIS-2005 scores were lower than those in AIS-1998 in the body regions of the head/neck (2.94 ± 1.08 vs. 3.40 ± 1.15, respectively, *p* < 0.001) and extremity (2.19 ± 0.56 vs. 2.24 ± 0.58, respectively, *p* < 0.001), but not in other body regions of the face, thorax, abdomen, and external organs (Table 2).

### 3.3. Comparison of Injury Characteristics between AIS-1998 and AIS-2005 According to the Injury Severity Score Strata

As a result of the difference between patients scored with the two AIS versions, several patients were newly classified into different ISS strata, defined as <16 (mild to moderate injury), 16–24 (severe injury), and >24 (critical injury). The percentage of patients classified into the ISS > 24 stratum (Table 3) were 4.7% (*n* = 351) and 3.5% (*n* = 266) for AIS-1998 and AIS-2005, respectively. For the critically injured patients, patients coded by AIS-2005 in the body regions of the extremity were significantly fewer than patients coded by AIS-1998. A significant difference in the proportion of patients between these two AIS versions in other body regions was not observed. Compared to AIS-1998, patients coded as critically injured by AIS-2005 had a significantly higher TRISS (0.33 ± 0.29 vs. 0.22 ± 0.41, respectively, *p* < 0.001), mortality rate (34.2% vs. 26.2%, respectively, *p* = 0.031), and rate of ICU admission (98.1% vs. 92.6%, respectively, *p* = 0.002). No significant difference in the hospital LOS was found between the groups.

As shown in Table 4, the percentage of patients classified into the ISS 16–24 stratum is 12.3% (*n* = 925) and 6.2% (*n* = 467) for AIS-1998 and AIS-2005, respectively, resulting in a decrease of nearly 50% of patients with an ISS 16–24 stratum coded by AIS-2005. Among this ISS stratum, there were significantly fewer patients coded by AIS-2005 in the body regions of head/neck than patients coded by AIS-1998, but there were more patients coded by AIS-2005 in the body regions of the face, thorax, abdomen, and extremity. Compared to AIS-1998, patients coded by AIS-2005 had a significantly higher TRISS (0.08 ± 0.12 vs. 0.01 ± 0.11, respectively, *p* < 0.001) and rate of ICU admission (85.2% vs. 67.0%, respectively, *p* < 0.001) and longer hospital LOS (median: 15 days vs. 9 days, respectively, *p* < 0.001). Although the mortality rate in AIS-2005 was higher than that in AIS-1998, the difference was marginal and nonsignificant (4.7% vs. 2.7%, respectively, *p* = 0.005).

As shown in Table 5, the percentage of patients classified into the ISS < 16 stratum is 83.0% (*n* = 6244) and 90.3% (*n* = 6787) for AIS-1998 and AIS-2005, respectively. Among this ISS stratum, there were significantly more patients coded by AIS-2005 in the body regions of the head/neck than those coded by AIS-1998, but there were fewer patients coded by AIS-2005 in the extremity than those coded by AIS-1998. Compared to AIS-1998, patients coded by AIS-2005 had a significantly higher TRISS (0.17 ± 0.02 vs. 0.00 ± 0.02, respectively, *p* < 0.001) and rate of ICU admission (13.8% vs. 10.4%, respectively, *p* < 0.001) than patients coded by AIS-1998. No significant difference in the rate of mortality and hospital LOS was observed between the groups.

Regarding patients with major trauma, which was defined as ISS >15 (i.e., the sum of patients in ISS 16–24 and >24 strata), the number of patients with major trauma in this study was 17.0% (*n* = 1,76) for AIS-1998 and 9.7% (*n* = 733) for AIS-2005. The mortality rate of patients with major trauma was significantly higher in AIS-2005 than that in AIS-1998 (15.4% vs. 9.1%, respectively, *p* < 000.1). Due to the number of patient deaths being nearly similar in those coded by AIS-1998 and AIS-2005 (*n* = 117 vs. n = 116), the significantly higher mortality rate was attributed to the remarkably reduced number of patients with major trauma coded by AIS-1998 and AIS-2005 (*n* = 1276 vs. *n* = 733). The distribution of AIS scores coded by AIS-1998 and AIS-2005 in the body regions of head/neck and extremity is shown in the Table 6. Of note, a coding with extremity AIS in AIS-1998 had been moved to a coding with AIS for the external injury in AIS-2005 in 15 patients.

## 4. Discussion

In this study, we revealed that the changes from AIS-1998 to AIS-2005 result in a significant decrease in the assessment of severity scores. The number of head/neck injuries classified as 16–24 was the key difference between AIS-1998 and AIS-2005. Among this ISS stratum, there were significantly fewer patients coded by AIS-2005 than patients coded by AIS-1998. Furthermore, critically injured patients with ISS > 24 coded by AIS-2005 had significantly higher mortality rate than patients coded by AIS-1998. Additionally, the rate of ICU admission was significantly higher for patients in all ISS strata after adapting AIS-2005 as the scoring system.

We found that AIS-2005 scores particularly in the body regions of the head/neck and extremity were significantly lower than AIS-1998 scores. These results were consistent with the reported literature. According to AIS-1998 version, significant specificity in the coding of traumatic brain injuries was not observed. The changes from AIS-1998 to AIS-2005 result in significant differences in severity scores, and most of the changes are observed in cerebellar and cerebral injuries [25]. For example, traumatic hypoxic brain injury secondary to systemic dysfunction was captured by AIS-2005 but not by AIS-1998. Additionally, subarachnoid hemorrhage with different sizes of the lesions had similar codes in AIS-1998, but this had been updated in AIS-2005. AIS-2005 differentiated injuries by size, resulting in additional code choices [9]. Furthermore, AIS-2005 captured fewer loss-of-consciousness cases due to changes in the criteria for coding concussive injury [25]. It had been estimated that in patients with traumatic brain injury, the mean AIS score coded by AIS-2005 was also significantly lower than that coded by AIS-1998 (2.85 (95% confidence interval, CI, 2.76–2.95) vs. 3.08 (95% CI, 2.99–3.18), *p* < 0.01) [25].

With the additional specificity of musculoskeletal/orthopedic injuries, a substantial increase in AIS-2005 codes in the upper and lower extremity from AIS-1998 had been noted. AIS-1998 did not code the upper extremity long bone fracture in the proximal, shaft, and distal sites, but AIS-2005 had added such a level of specificity [9]. The codes and descriptions specifically for humerus shaft fracture of the upper extremity and bimalleolar fractures were expanded from AIS-1998 to AIS-2005 [9]. Furthermore, some long bone fractures were listed as AIS 3 in AIS-1998 but downgraded to a severity of AIS 2 in AIS-2005 [9].

Regarding the decreased number of patients categorized as having major trauma under the definition of ISS > 15, this study presented similar results to that reported by Salottolo et al. [16] and Palmer et al. [17]. With the adaptation of AIS-2005, a decrease in the number of patients with major trauma ranging from 17% [26] to 22% [27] has been observed based on the Victorian major trauma criteria. In this study, the number of patients with major trauma for AIS-2005 was 9.7% (*n* = 733), with a 42.5% decrease in the number of patients experiencing major trauma from 17.0% (*n* = 1276) for AIS-1998. In our data, the mortality rate of patients with major trauma with an ISS > 15 increased from 9.1% in the patients coded by AIS-1998 to 15.4% in the patients coded by AIS-2005. Based on the ISS calculation, an ISS > 15 indicated a severe-to-critical injury, which was associated with a 10% mortality rate from the Major Trauma Outcome Study population [28,29]. This is no longer the case when considering the significant improvements in trauma management in the past decades. Several AIS codes have had their AIS severity scores reduced between AIS-1998 and AIS-2005 because with the advancements in trauma management, some injuries are easily treated. Although the severities of AIS-2005 may more accurately reflect the medical practices today, according to this study, the increase in mortality rate by adapting the AIS-2005 rather than the older AIS-1998 version should be taken into consideration, specifically when comparing the outcome of trauma management among different times or different hospitals. A direct comparison of the measurements that are generated from these two AIS versions can produce misleading results. Additionally, the most recent AIS revision, AIS-2015 from the Association for the Advancement of Automotive Medicine, had already been used and considered as an option in the US National Trauma Data Standard for 2019 [30]. Therefore, it is also necessary to continue to evaluate this essential tool and the scores that are derived from it.

This study has some limitations. First, the study involved trauma patients in one hospital only; hence, the generalization of the results to other hospitals or regions should be further assessed. Second, the patients who were declared dead at the scene of the injury were excluded, and only in-hospital mortality was included in the registered trauma database, which may result in a selection bias in the assessment of mortality rate. Finally, the indications or results of trauma management, surgical intervention, and indications for ICU admission for these patients were not considered in the analysis of this study, and we can only assume such options for these patients did not differ.

## 5. Conclusions

In this study, we revealed that the adaptation of the AIS-2005 from the AIS-1998 had resulted in a significant decrease in the measurement of severity scores. The significant decreases in AIS scores are in the body regions of the head/neck and extremity. As a consequence, critically injured patients who had ISS >24 coded by AIS-2005 had a higher mortality rate than patients coded by AIS-1998.

## Figures and Tables

**Table 1:** Characteristics and outcomes of the study population coded by Abbreviated Injury Scale (AIS)-1998 and AIS-2005.

AIS Version	AIS-1998	AIS-2005
Patient Number	7520	
Age (Years)	49.5 ± 22.8	
Gender, *n* (%)		
Male	4145 (55.1)	
Female	3375 (44.9)	
Mortality, *n* (%)	135 (1.8)	
ICU Admission, *n* (%)	1596 (21.2)	
Hospital LOS, days (Q1–Q3)	6 (3–10)	
AIS, *n* (%)		
Head/Neck	1528 (20.3)	1528 (20.3)
Face	857 (11.4)	857 (11.4)
Thorax	928 (12.3)	930 (12.4)
Abdomen	431 (5.7)	431 (5.7)
Extremity	5162 (68.6)	5147 (68.4)
External	1070 (14.2)	1083 (14.4)
ISS	8.3 ± 7.1	7.5 ± 6.3 *

AIS = Abbreviated Injury Scale; ICU = intensive care unit; ISS = Injury Severity Score; LOS = length of stay; Q1 = the first quartile; Q3 = the third quartile; * indicates significant when compared with AIS-1998.

**Table 2:** Characteristics and outcomes of the study population coded by Abbreviated Injury Scale (AIS)-1998 and AIS-2005.

AIS of Body Region	AIS-1998	AIS-2005	*p*
Head/Neck	3.40 ± 1.15	2.94 ± 1.08 *	<0.001
Face	1.79 ± 0.43	1.77 ± 0.45	0.207
Thorax	2.65 ± 0.87	2.60 ± 0.78	0.203
Abdomen	2.68 ± 0.94	2.69 ± 0.94	0.971
Extremity	2.24 ± 0.58	2.19 ± 0.56 *	<0.001
External	1.22 ± 0.58	1.21 ± 0.58	0.888

AIS = Abbreviated Injury Scale; The continuous data are expressed as mean ± standard deviation; * indicates significant when compared with AIS-1998.

**Table 3:** Characteristics and outcomes of the patients with Injury Severity Score >24 coded by Abbreviated Injury Scale (AIS)-1998 and AIS-2005.

ISS> 24	AIS-1998 *n* = 351	AIS-2005 *n* = 266	*p*
Age (Years)	51.3 ± 21.0	50.9 ± 20.8	0.791
Gender, *n* (%)			0.435
Male	250 (71.2)	197 (74.1)	
Female	101 (28.8)	69 (25.9)	
Mortality, *n* (%)	92 (26.2)	91 (34.2) *	0.031
ICU Admission, *n* (%)	325 (92.6)	261 (98.1) *	0.002
Hospital LOS, days (Q1–Q3)	15 (8–26)	16 (7–28)	0.157
AIS, n (%)			
Head/Neck	289 (82.3)	208 (78.2)	0.198
Face	72 (20.5)	50 (18.8)	0.596
Thorax	171 (48.7)	121 (45.5)	0.426
Abdomen	91 (25.9)	77 (28.9)	0.404
Extremity	177 (50.4)	111 (41.7) *	0.032
External	30 (8.5)	24 (9.0)	0.836
TRISS	0.22 ± 0.41	0.33 ± 0.29 *	<0.001

AIS = Abbreviated Injury Scale; ICU = intensive care unit; ISS = Injury Severity Score; LOS = length of stay; TRISS = Trauma Injury Severity Score; * indicates significant when compared with AIS-1998.

**Table 4:** Characteristics and outcomes of the patients with Injury Severity Score 16–24 coded by Abbreviated Injury Scale (AIS)-1998 and AIS-2005.

ISS 16–24	AIS-1998 *n* = 925	AIS-2005 *n* = 467	*p*
Age (years)	54.8 ± 22.2	50.4 ± 22.0 *	<0.001
Gender, *n* (%)			0.075
Male	575 (62.2)	313 (67.0)	
Female	350 (37.8)	154 (33.0)	
Mortality, n (%)	25 (2.7)	22 (4.7)	0.050
ICU admission, n (%)	620 (67.0)	398 (85.2) *	<0.001
Hospital LOS, days (Q1–Q3)	9 (5–16)	15 (10–24) *	<0.001
AIS, *n* (%)			
Head/Neck	752 (81.3)	346 (74.1) *	0.002
Face	179 (19.4)	121 (25.9) *	0.005
Thorax	202 (21.8)	176 (37.7) *	<0.001
Abdomen	106 (11.5)	112 (24.0) *	<0.001
Extremity	288 (31.1)	242 (51.8) *	<0.001
External	79 (8.5)	41 (8.8)	0.881
TRISS	0.01 ± 0.11	0.08 ± 0.12 *	<0.001

AIS = Abbreviated Injury Scale; ICU = intensive care unit; ISS = Injury Severity Score; LOS = length of stay; TRISS = Trauma Injury Severity Score; * indicates significant when compared with AIS-1998.

**Table 5:** Characteristics and outcomes of patients with Injury Severity Score <16 coded by Abbreviated Injury Scale (AIS)-1998 and AIS-2005.

ISS <16	AIS-1998 *n* = 6244	AIS-2005 *n* = 6787	*p*
Age (Years)	48.6 ± 22.9	49.4 ± 23.0	0.056
Gender, *n* (%)			0.658
Male	3320 (53.2)	3635 (53.6)	
Female	2924 (46.8)	3152 (46.4)	
Mortality, *n* (%)	18 (0.3)	22 (0.3)	0.712
ICU Admission, *n* (%)	651 (10.4)	937 (13.8) *	<0.001
Hospital LOS, Days (Q1–Q3)	5.0 (3.0–9.0)	5.0 (3.0–9.0)	0.755
AIS, *n* (%)			
Head/Neck	487 (7.8)	974 (14.4) *	<0.001
Face	606 (9.7)	686 (10.1)	0.443
Thorax	555 (8.9)	633 (9.3)	0.385
Abdomen	234 (3.7)	242 (3.6)	0.580
Extremity	4697 (75.2)	4794 (70.6) *	<0.001
External	961 (15.4)	1021 (15.0)	0.581
TRISS	0.00 ± 0.02	0.17 ± 0.02 *	<0.001

AIS = Abbreviated Injury Scale; ICU = intensive care unit; ISS = Injury Severity Score; LOS = length of stay; TRISS = Trauma Injury Severity Score; * indicates significant when compared with AIS-1998.

**Table 6:** Distribution of AIS scores coded by Abbreviated Injury Scale (AIS)-1998 and AIS-2005 in the body regions of head/neck and extremity.

		**AIS for Head/Neck**				
		AIS-1998				
	**AIS in different versions**	Minor/Mode (AIS = 1 + 2)	Serious (AIS = 3)	Severe (AIS = 4)	Critical (AIS = 5)	Total
AIS-2005	Minor/Moderate (AIS = 1 + 2)	330 (21.6%)	118 (7.7%)	2 (0.1%)	0 (0.0%)	450 (29.5%)
	Serious (AIS = 3)	0 (0.0%)	128 (8.4%)	573 (37.5%)	0 (0.0%)	701 (45.9%)
	Severe (AIS = 4)	0 (0.0%)	0 (0.0%)	216 (14.1%)	6 (0.4%)	222 (14.5%)
	Critical (AIS = 5)	0 (0.0%)	0 (0.0%)	2 (0.1%)	152 (9.7%)	154 (10.1%)
	Total	330 (21.6%)	246 (16.1%)	793 (51.9%)	158 (10.3%)	1528 (100%)
		**AIS for Extremity**				
		AIS-1998				
	**AIS in different versions**	Minor/Mode (AIS = 1 + 2)	Serious (AIS = 3)	Severe (AIS = 4)	Critical (AIS = 5)	Total
AIS-2005	Minor/Moderate (AIS = 1 + 2)	3544 (68.7%)	220 (4.3%)	1 (0.02%)	0 (0.0%)	3765 (72.9%)
	Serious (AIS = 3)	4 (0.08%)	1360 (26.3%)	0 (0.0%)	0 (0.0%)	1364 (26.4%)
	Severe (AIS = 4)	0 (0.0%)	0 (0.0%)	18 (0.3%)	0 (0.0%)	18 (0.3%)
	Critical (AIS = 5)	0 (0.0%)	0 (0.0%)	0 (0.0%)	0 (0.0%)	0 (0.0%)
	Total	3548 (68.7%)	1580 (30.6%)	19 (0.4%)	0 (0.0%)	5162 (100%)
